# Recent advances in electrochemical sensors and biosensors for monitoring drugs and metabolites in pharmaceutical and biological samples

**DOI:** 10.5599/admet.1709

**Published:** 2023-05-01

**Authors:** Charaf Laghlimi, Abdelaziz Moutcine, Abdelilah Chtaini, Jalal Isaad, Adil Soufi, Younes Ziat, Hassan Amhamdi, Hamza Belkhanchi

**Affiliations:** 1 ERCI2A, FSTH, Abdelmalek Essaadi University, Tetouan, Morocco; 2 Molecular Electrochemistry and Inorganic Materials Team, Faculty of Science and Technology of Beni Mellal, Sultan Moulay Slimane University, Morocco; 3 Laboratory of Engineering and Applied Technologies, Higher School of Technology, Sultan Moulay Slimane University, Beni Mellal, Morocco; 4 Applied Chemistry Team, FSTH, Abdelmalek Essaadi University, Tetouan, Morocco

**Keywords:** Pharmaceuticals and drugs, screen printed carbon electrodes, glassy carbon electrodes, carbon nanotubes

## Abstract

Various applications of electrochemical sensors and biosensors have been reported in many fields. These include pharmaceuticals, drug detection, cancer detection, and analysis of toxic elements in tap water. Electrochemical sensors are characterised by their low cost, ease of manufacture, rapid analysis, small size and ability to detect multiple elements simultaneously. They also allow the reaction mechanisms of analytes, such as drugs, to be taken into account, giving a first indication of their fate in the body or their pharmaceutical preparation. Several materials are used in the construction of sensors, such as graphene, fullerene, carbon nanotubes, carbon graphite, glassy carbon, carbon clay, graphene oxide, reduced graphene oxide, and metals. This review covers the most recent progress in electrochemical sensors used to analyze drugs and metabolites in pharmaceutical and biological samples. We have highlighted carbon paste electrodes (CPE), glassy carbon electrodes (GCE), screen-printed carbon electrodes (SPCE) and reduced graphene oxide electrodes (rGOE). The sensitivity and analysis speed of electrochemical sensors can be improved by modifying them with conductive materials. Different materials used for modification have been reported and demonstrated, such as molecularly imprinted polymers, multiwalled carbon nanotubes, fullerene (C60), iron(III) nanoparticles (Fe_3_O_4_NP), and CuO micro-fragments (CuO MF). Manufacturing strategies and the detection limit of each sensor have been reported.

## Introduction

Pharmacokinetics aims to determine a drug's pathway from administration to excretion [1]. For clinical diagnosis, there is a need for information on how medicines work in the human body and the dose that should be administered [2]. It is, therefore, necessary to quantify drugs in physiological fluids such as serum, urine, sweat and saliva, as well as in pharmaceutical tablets. Several sensitive and accurate methods have been used to quantify and detect different types of drugs, such as spectrophotometry [3,4], fluorimetry [3], RP-HPLC [4], HPLC-diode array detector, TLC-densitometric methods [5] and capillary zone electrophoresis [6]. However, these techniques are very expensive and require the use of expensive solvents, specialised technicians, long sample preparation times and expensive instrumentation, all of which increase the cost and duration of the analysis [2]. For this reason, the pharmacokinetic community strives to provide alternative methods of drug surveillance that are easy, inexpensive, sensitive and precise [7]. In the last decades, electrochemical sensors have become an interesting alternative technique due to their high capacity to analyse drugs in different physiological fluids [8-10]. Graphene has considerable sensitivity and selectivity and often has a large potential window to accommodate the redox process involved. It also has a significant electron transfer process due to its planar sites and the presence of σ and π bonds, making it an attractive material for sensor manufacture [11,12]. Modification of the carbon paste electrode offers high sensitivity, low cost and rapid analysis without any pre-treatment [13-21]. The modification is an effective way to reduce the overpotential and increase the sensitivity of the sensors [22]. Various electrodes based on carbon as a conductor have been used for the electroanalysis of drugs [23,25].

In this review, we report on a new advance in carbon graphite-based electrochemical sensors used to analyse different types of drugs in pharmaceutical tablets and a variety of physiological fluids such as serum, blood, sweat and urine. The different modifiers used to improve sensor performance and increase the electroactive surface area are studied. Sensitivity, detection limit and method of preparation have been highlighted to provide comprehensive information on all these techniques and their use in drug analysis.

## Electrodes based on modified carbon paste for detecting various drugs and metabolites

Carbon paste electrodes (CPE) have a very large electroactive surface area, which can be renewed for a variety of applications. In addition, it has low ohmic resistance and high stability, reproducibility and lifetime, allowing it to analyse various drugs, revealing the oxidation and reduction processes involved due to its wide potential range. The sensitivity and speed of analysis of CPE can be significantly improved by modifying them with conductive materials. Different types of modifiers have been used. The modified CPE has been used to examine various drugs in various samples, including human urine, pharmaceutical formulations, plasma and serum.

**Figure 1.**
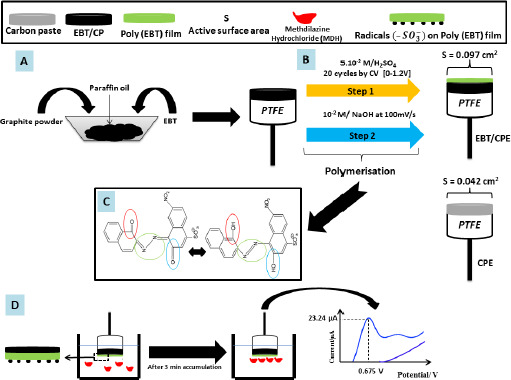
Schematic of A) EBT/CPE preparation, B) polymerisation steps of modified CPE, C) proposed mechanism of eriochrome black T (EBT) polymerisation and D) electrochemical detection of MDH by EBT/CPE. Carried out at the base of the reference [26] with an order License ID of 1346497-1

The carbon paste electrode modified with poly(EBT) [26] exhibits very high electrocatalytic and semiconducting for detecting methdilazine hydrochloride (MDH), an antihistamine drug, in Dilosyn syrup and human urine using the SWV method (Figure 1). The MDH oxidation peak is observed at 0.675V with higher intensity compared to unmodified CPE. The surface area of poly-EBT/CPE is calculated to be 0.097 cm^2^, which is 2.30 times higher than that of CPE. The developed sensor shows good accuracy in both media (average recovery is 98.14 % in Dilosyn syrup and 97.4 % in human urine). The developed sensor has a low LOD of the order of 10^-8^ mol l^-1^ in the range of 0.1-50 μmol l^-1^ compared to other methods such as visible spectrophotometry (3.23 μmol l^-1^) [27], ultra-high performance reversed phase chromatography (0.254 μmol l^-1^ ) [28] and spectrophotometric method (1.62 μmol l^-1^ ) [29].

The researchers [30] reported that CPE/nanozeolite type X outperformed CPE/nanozeolite type A with a LOD of 0.2 μmol l^-1^ for paracetamol and 8 mmol l^-1^ for epinine.

A recent study described the analysis of the antifungal agent ketoconazole (KTC) in pharmaceutical and urine samples using Ce-BTC MOF/IL/CPE (*cf.* Table 1) [31]. The modifiers are synthesised according to the method of Liu [32]. The electrochemical behaviour is studied by chronoamperometry (CA), differential pulse voltammetry (DPV), cyclic voltammetry (CV) and linear sweep voltammetry (LSV). The LOD of Ce-BTC MOF/IL/CPE is 0.04 μmol l^-1^ in the range of 0.1-110.0 μmol l^-1^ . The sensitivity is found to be 0.1342 μA μmol^-1^ l.

J. Zoubir *et al.* [33] used silver nanoparticles to modify carbon graphite by electrodeposition. The fabricated sensor was used to detect metronidazole in milk and tap water with a detection limit of 0.206 μmol l^-1^ in the range 1-1000 μmol l^-1^ .

O. Vajdle *et al.* [34] used the drop coating method to modify CPE with gold nanoparticles. AuNPs/CPE were used to analyse four macrolide antibiotics by SWV. AuNPs/CPEs are used for azithromycin (AZI) detection in Hemomycin® by the SWV method. The oxidation peak of the AZI is identified at 0.77 V on the AuNPs/CPE, while the roxithromycin (ROX) peak is found at 0.65 V by CPE in the Runac®. Both electrodes were found to be valid by comparison with HPLC-DAD measurements.

On the other hand, nitric acid and sulphuric acid are used to increase -OH and -COOH groups on the surface of MWCNTs, which increases the surface area of the material [35]. In addition, the numerous carboxyl groups inhibit π-π interactions, which leads to a decrease in the adsorbency of MWCNTs [36]. Ofloxacin (OFX) adsorbs on MWCNT by binding its fluorine group to the -OH group of the CNT [37-40]. Flake graphite (FG) is used to increase the conductivity of MWCNTs weakened by the adsorption of compounds owing to its high degree of crystallisation. For this reason, M. Elfiky *et al.* [41] prepared an electrode by mixing graphite powder with FG and MWCNTs. The sensor has a larger electroactive area of 2.08 cm^2^ compared to the unmodified CPEs, with only 1.14 cm^2^. This finding is due to cracks on the irregular compact layers of the electrode. The electrode presented by the [10%FG/5%MW] CPE shows excellent performance in the analysis of ofloxacin in a commercial formulation (Ofloxacin® tablets) and in human urine samples with a LOD of 0.18 nmol l^-1^. OFX peak is observed at 0.85V by SW-AdAS

The analysis of AZI, an alkaline chemical, by HPLC presents a difficulty due to its adsorption on Si-OH chromatographic materials [42]. Therefore, electrochemiluminescence detection is considered an alternative method for the detection of this antibiotic due to its sensitivity and high productivity [43,44]. Electrochemiluminescence (ECL) is based on the redox process involved in the formation of excited states that are able to emit light. The concept of molecularly imprinted polymers (MIP) is based on imprinting a molecular cavity on the surface of the polymer using a template molecule, which is then removed while the polymer is still imprinted. This MIP can then be used to capture and detect a target molecule similar to the template molecule and specifically binds to a functional group in the cavity in the same way that an antibody binds to its antigen. There are many ways to make MIPs, such as soft lithography applied to giant molecules. L. Hu *et al.* [45] successfully analysed azithromycin (AZI) in urine and serum samples using MIP/CP ECL sensor, with MIP used as a recognisor. The oxidation peak of the amino groups of AZI [31] is observed at 0.8 V with LOD of 23 pmol l^-1^ in the range of 0.10-400 nmol l^-1^. The analytical results show the MIP/CP ECL sensor and HPLC agreement.

Clay has attractive characteristics, like high specific surface area and cation exchange capacity [46,47]. Bakary Tigana Djonse Justin *et al.* [48] recently fabricated a titanium dioxide modified carbon clay paste electrode (CPEA/TiO_2_/UV) that allows the analysis of ascorbic acid in pharmaceutical tablets using CV.The sensitivity of the electrode is enhanced by photoactivation of TiO_2_ by light irradiation (with a 100 W lamp), which produces electron (e)/hole (h+) pairs upon absorption of appropriate light energy [49]. The detection limit is 0.732 μmol l^-1^in the range of 0.15-0.850 μmol l^-1^ .

V. Vinoth *et al.* [50] modified a graphite carbon electrode with CuO microflowers (MFs) by solubilising them in Nafion to facilitate the modification. This electrode was named CuO MFs/Nafion/GC. The CuO MFs/Nafion/GC exhibited perfect electrocatalytic properties, selectivity in the presence of interfering molecules, stability and reproducibility. The amperometric analysis of glucose showed a sensitivity of 3.1 μA μmol^-1^ l cm^-2^ and LOD of 6.48 μmol l^-1^ between 10 to 120 μmol l^-1^ at +500 mV. This detection limit is very good compared to other sensors used for the same purpose such as NiO-SWCNT (907 μmol l^-1^ ) [51]. Table 1 shows the different types of modified CPEs as well as the drugs detected and their detection medium, the limit of detection (LOD), concentration range and other characteristics.

The year of publication is indicated in each table in order to highlight the most recent publications and make them directly accessible to the reader.

## Electrodes based on modified glassy carbon for detecting various drugs and metabolites

Glassy carbon is a type of carbon with an amorphous structure like glass and ceramics. Glassy carbon is an interesting material for electrochemical sensors because of its chemical resistance, low density and low electrical resistance. Glassy carbon electrodes (GCE) have also played an important function in electrochemical sensors and biosensors for drug analysis. The electron transfer rate at the GCE surface can be increased by various modifiers for the detection of medicines in diverse media.

The GCE/ZnO@NDCS/GOx is an enzymatic biosensor used to analysis glucose in serum (*cf.* Table 2) [54]. The biosensor had a reproducible sensitivity of 231.7 μA mol^-1^ l cm^-2^ with a LOD of 6.3 μmol l^-1^ at 0.57 V. The sensor selectivity is demonstrated via the addition of some interfering potentials such as ascorbic acid, dopamine, fructose, uric acid and mannose (Man) with glucose, resulting in only a small increase in current for each species. Due to the easy transfer of electrons through this biosensor, the response time is very short (<3 s). The biosensor is well suited for glucose analysis with recovery between 99.73-100.14 % and retains about 95.3 % of the initial response during 50 days of storage in human blood.

Acyclovir (ACV) (9-(2-hydroxyethoxymethyl)guanine) is a drug largely employed for the therapy of viral skin infections and neuritis [55-57]. Overdoses lead to adverse effects in patients [58,59]. The FeMoO_4_ compound is able to catalytically fix nitrogen [60] due to its interesting electrocatalytic property [61]. It also has interesting redox kinetics, making it a desirable material for electrode formation. Recently, Y. Wei *et al.* [62] prepared a sensor using graphene oxide composites loaded (ultrasonically) with ferrous molybdate (FeMoO_4_) for the analysis of ACV in pharmaceuticals by LSV. The oxidation peak of ACV is observed at 1.1 V. FeMoO_4_-GO/GCE and has two linearity intervals (0.1-10 and 10-100 μmol l^-1^ ) with a LOD of 20 nmol l^-1^. The reaction of ACV on the developed electrode is diffusion controlled with 2e^-^ and 2H^+^ transferred. The active surface area of FeMoO_4_-GO/GCE is 1.59 times larger than that of FeMoO_4_/GCE, which clearly shows that the FeMoO_4_-GO improves the performance of the sensor.

**Figure 2.**
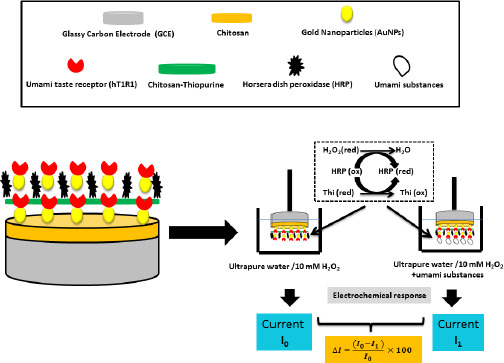
Schematic of the multilayer modification of the GCE to study the electrochemical response of the human umami taste receptor hT1R1 towards umami substances such as sodium glutamate, disodium inosinate and disodium guanylate. Carried out at the base of the reference [63] with an order License ID of 1346500-1

Y. Huang *et al.* [63] fabricated a biosensor to study the human umami taste receptor (hT1R1) and the umami substances, such as monosodium glutamate (MSG), using a multilayer material to modify the glassy carbon electrode (Figure 2). During the fabrication of this electrode, a human umami taste receptor (hT1R1) was attached to the layers formed by the AuNPs [64]. Horseradish peroxidase (HRP) is used for direct electron transfer to the multilayer material formed [65]. The researchers suggest that hT1R1 is likely a receptor used by the body to sense nitrogen, opening up a new way of studying nutrient and drug adsorption.

**Figure 3.**
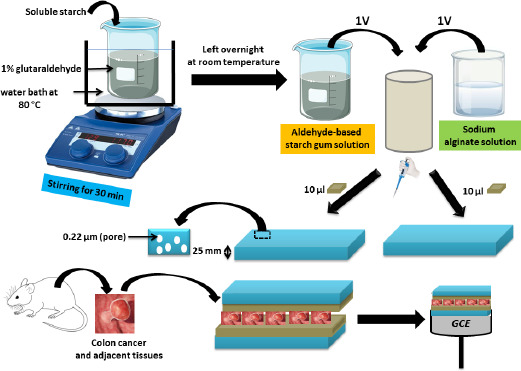
Schematic of the multilayer modification of GCE to study the detection kinetics of C and N nutrient receptors. Based on the reference [66]

In 2023, the same researchers [66] created another biosensor by attaching colon cancer and adjacent tissues to GCE to visualise the kinetics of responding to C and N nutrient receptors such as glucose and sodium lactate. In order to do this, they mixed solutions of starch gum with an aldehyde base and sodium alginate, which were spread over two microporous polycarbonate membranes into which the colon tissues were placed to build a layered assembly aligned to the GCE (Figure 3). Researchers found that the cells had different sensitivities to lactate, suggesting the possibility of using this nutrient to treat colon cancer. Colon cancer tissue is insensitive to lactate, whereas adjacent tissue is sensitive.

CoCo_2_O_4_ nanorods embedded in hexagonal boron nitride are used to modify the GCE for ronidazole determination [67]. The electrode synthesis presents a nano-LOD of 3 nmol l^-1^ between 0.01 and 1345 μmol l^-1^and a higher sensitivity of 5.845 μA μmol^-1^ l cm^-2^ using DPV. This sensitivity is explained by the enhanced adsorption and transport of mass ions via the formation of aggregates of hexagonal boron nitride (h-BN) and spinel cobalt oxide nanorods (CoCo_2_O_4_ NRs). CoCo_2_O_4_ NRs are used for their low cost, good stability and other properties. The h-BN, formed from boron and nitrogen, has a structure similar to graphene [68], which makes it an electroactive material [69,70].

Methotrexate (MTX) is a drug that inhibits the growth of tumour cells. It is applied for the treatment of certain types of cancer, notably breast cancer [71] and pulmonary cancer [72]. GO-Nafion-GCE sensor is constructed by dispersing graphite oxide in a solution containing Nafion-ethanol and then using it to modify the GCE [73]. Modified electrode stabilisation is achieved by using a CV of 0.1 V/s and in the potential between 0.5 and 1.2 V. GO-Nafion-GCE is able to detect methotrexate in MTX injection and urine with a LOD of 9 nmol l^-1^ between 0.4 and 20 μmol l^-1^ , that is close to the LOD of detection of the same drug by 3DPG-CNT/GCE [74]. The electrolyte solution used for the analysis of MXT is perchloric acid (0.03 mol l^-1^). A mixed adsorption-diffusion phenomenon controls the reaction process of MXT on the electrode surface.

Dopamine (DA) and paracetamol (PA) detection in synthetic urine was performed using a GCE-ERGO/polyCoTAPc electrode [75]. First, GCE was modified by electrochemically reduced graphene oxide (ERGO), which was simultaneously reduced and deposited from graphene oxide (GO). Finally, GCE-ERGO/polyCoTAPc was formed by electropolymerisation of cobalt (II) tetra-amino phthalocyanine. The developed electrode sensitivity was 1.32 μA mol^-1^ l cm^-2^ for PA and 8.39 μA mol^-1^ l cm^-2^ for DA. LOD is 0.10 μmol l^-1^ for PA and 0.095 μmol l^-1^ for DA using DPV.

The modification of GCE by QDs-P6LC-PEDOT:PSS allowed the amoxicillin (AMX) analysis in synthetic urine, whole milk and pharmaceuticals [76]. AMX oxidation peak is 0.88V and the reaction process on QDs-P6LC-PEDOT:PSS/GCE is irreversible controlled by diffusion process with an equal number of proton and electron exchange. The researchers found that the alkaline medium facilitates the deprotonation of AMX during the oxidation reaction. Furthermore, the detection limit is found to be 50 nmol l^-1^ in the range 0.90-69 μmol l^-1^ .

To form graphene (GR)-ZnO/GCE, X. Yue *et al.* [80] first polished the surface of GCE with a suspension of Al_2_O_3_ powder on a polishing cloth. Then, 8 μl of GR-ZnO nanocomposite suspension is used to modify the glassy carbon electrode. GR-ZnO/GCE was used for the simultaneous detection of sulfamethoxazole (SMZ) and trimethoprim (TMP) in urine and serum. SMZ and TMP oxidation peaks were successfully located at the *E*_p_ (SMZ) = 0.85 V and *E*_p_ (TMP) = 1.06 V by the DPV method, with a slight shift in peak potentials compared to those observed on the GCE. The researchers explained this by the ability of the GR to enhance ZnO nanorods conductivity and the reciprocal ability of the ZnO nanorods to avoid aggregation of the GR by reducing the van der Waals force. SMZ and TMP oxidation reactions are irreversible. They are controlled by adsorption. SMZ LOD is 0.4 μmol l^-1^ between 1-220 and 0.3 μmol l^-1^of TMP in the range of 1-180 μmol l^-1^ . The same drugs have been detected by GCE modified with GO and Ag nanoparticles (GC/rGO-AgNP) [81] using DPV with LOD (SMZ) = 0.6 μmol l^-1^ and LOD (TMP) = 0.4 μmol l^-1^ between 1.0 and 10.0 mmol l^-1^. In addition, SMZ and TMP exhibit irreversible oxidation peaks with Δ*E*_TMP-SMZ_= 1.14-0.92 = 0.22V.

A further study has shown that the nickel ferrite/rGO (NiFe_2_O_4_/rGO) film is an excellent modifier of the GCE. Clenbuterol was analyzed in pig urine samples using the electrode produced [82]. The LOD is 0.17 μmol l^-1^ between 0.99 and 18.03 μmol l^-1^ using DPV.

**Figure 4.**
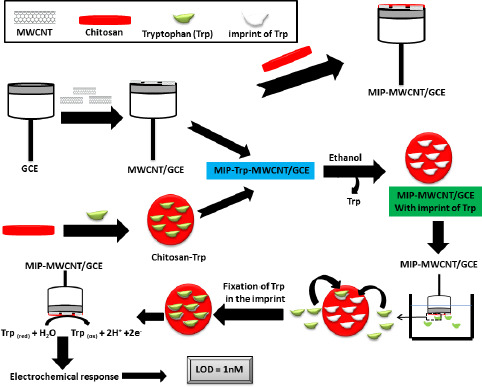
Schematic of MIP-MWCNT/GCE preparation and electrochemical detection of Tryptophan. Based on the reference [85]

Due to the excellent selectivity of chitosan [83] and the high selectivity and ease of preparation of the MIP method [84], Y. Wu *et al.* [85] fabricated a sensor called MIP-MWCNTs/GCE for the detection of tryptophan (Trp), a possible cause of schizophrenia [86], by depositing a printed chitosan film on the MWCNT pre-modified GCE surface. This pre-modification with MWCNTs aims to enhance the response of molecularly imprinted polymer electrodes [87]. MWCNTs are a type of carbon nanotube with highly active sites due to the presence of more concentric tubes [88], which gives them high adhesive activity and good conductivity. Y. Wu *et al.* [85] demonstrated hydrogen bonding between chitosan and Trp using Fourier transform infrared (FTIR) spectroscopy. The extraction reagent (ethanol) used during the extraction process causes the chitosan film to deflate and etch, forming a porous structure capable of efficiently binding Trp molecules to these imprinted sites (Figure 4). The oxidation process of Trp involves the exchange of an identical number of e^-^ and H^+^. Analysis of Trp in human serum by MIP-MWCNTs/GCE showed a very encouraging recovery between 96.5 and 102.5 %. In the presence of several interferents, the Trp peak is very intense compared to the other substances. The MIP-MWCNTs/GCE sensor has a low LOD (1 nmol l^-1^) compared to the GCE sensor modified with AuNP and MWCNTs [89].

Daunorubicin is an anticancer drug [90] that requires dose control [91] to avoid adverse effects, including cardiac arrest. H. Karimi-Maleh *et al.* [92] attempted to develop a glassy carbon electrode modified with a nanocomposite of Pt/SWCNTs and ds-DNA (biorecognition) to eventually form ds-DNA/Pt/SWCNTs/GCE. DVP shows a positive displacement of the daunorubicin oxidation peak (from 847 to 882 mV), confirming the mutual intercalation reaction of ds-DNA (guanine base) and daunorubicin. Furthermore, the equilibrium constant of the association is 5.044×10^3^ mol^-1^ l with a nanometre LOD (1.0 nmol l^-1^) over a concentration between 4.0 nmol l^-1^ and 250.0 μmol l^-1^.

Using the Fe_3_O_4_/MWCNT/GCE electrode, T. Bhengo *et al.* [93] obtained oxidation peaks of sulfamethoxazole and trimethoprim at 910 and 1120 mV, respectively, with detection limits of 11.0 nmol l^-1^ for SMZ and 21.0 nmol l^-1^ for TMP using the DPV method. Similarly, Rajasekhar Chokkareddy *et al.* [94] deposited IL-f-ZnONPs@MWCNTson the clean surface of GCE by dispersing them in dimethylformamide (*cf.* Table 2).To prepare a solution containing SMZ, the researchers crushed two Sandoz Co-Trimoxazole (Pharmaceuticals) tablets and dissolved part of the powder in methanol. After shaking, the solution was refined and diluted with PBS (pH 6.0). The resulting solution was then added to the pharmaceutical urine samples. The cyclic voltammetry oxidation peak of the SMX was found to be at 620 mV. The reaction process on IL-f-ZnONPs@MWCNTs@GCE is diffusion controlled and the detection limit is 0.1 ng/ml in the range of 0.1-10 ng ml^-1^. In addition, Ag-MWCNT/MTOAC/GCE [95] was successfully used to detect SMX in Co-trimoxazole (mg per tablet), Cotrim paediatric and human urine by DPV. The samples were diluted with phosphate solution to reduce the matrix effect and then introduced into the electrochemical cell. The irreversible oxidation of the SMX is observed at +0.85 V and involves the exchange of an equal number of e^-^ and H^+^ and is controlled by adsorption.

On the other hand, the simultaneous analysis of ascorbic acid (AA), dopamine and uric acid in human urine was investigated using a GCE-based sensor modified with 2D titanium carbide nanoplatelets (MXene) [96]. All three analytes showed remarkably distinct oxidation peaks. AA was observed at 0.001 V, while dopamine and uric acid were at 210 mV and 330 mV, respectively. It should be noted that 2D titanium carbide (MXene) nanoplatelets are mentioned in the literature as having a high conductivity of 9880 S cm^-1^ [102], higher than that of graphene. In particular, MXene groups, such as fluor, oxygen and hydroxyl, increase its hydrophilicity and allow rapid access to the analyte. It is therefore used as an ideal material for sensors [103].

Table 2 shows the different types of modified GCEs as well as the drugs detected and their detection medium, the limit of detection (LOD), concentration range and other characteristics.

## Electrodes based on modified graphene oxide for detecting various drugs and metabolites

Graphene oxide reduced (rGO) is obtained by thermal, electrochemical or chemical treatment of graphene oxide using substances such as NaBH_4_ or aluminium powder. This reduction removes oxidised functional groups (which increases the sensitivity of the GO and makes it insulating) and creates a defect structure (active sites) characteristic of rGO. These properties have enabled rGO to have high electrochemical activity compared to graphene and graphene oxide.

F. Zhou *et al.* [104] successfully fabricated a Nafion/GOx/Au-ZnO/rGO/ITO electrode. The researchers found that the rate and efficiency of electron transfer were augmented by the deposition of AuNPs and UV irradiation, allowing a significant increase in glucose sensitivity (*cf.* Table 3). LOD of this sensor is 0.2 μmol l^-1^ by amperometric method between 0 and 9.5 mmol l^-1^ with a higher Michaelis-Menten constant (15.54 mmol l^-1^) than that of non-UV irradiated Nafion/GOx/Au-ZnO/rGO/ITO. The sensors have exceptional detection accuracy for blood glucose measurement.

Regorafenib (REG) or Regonix is an anticancer drug used in several types of cancer [105,106]. Monitoring the concentration of REG in serum or blood seems to be of great interest as doses above certain limits can cause adverse effects on vital organs of the body [107]. REG is detected in real samples by Pd-Ru/rGO using the DPV technique [108] (*cf.* Table 3). The acidic graphene neutrality is obtained from pomegranate peel extract (PPE). In fact, the redox current peak is linear with s*v* (*v* = potential sweep rate), indicating that the REG reaction occurring at Pd-Ru/rGO is diffusion controlled, with a constant heterogeneous charge transfer rate equal to *K*^0^ = 2.29 s^-1^, calculated based on Laviron's equation (*cf.* Eq. (1), (2) and (3)) [109]. Furthermore, the REG oxidation involves the exchange of 2e^-^ and 2H^+^.


(1)

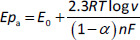




(2)

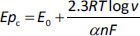




(3)

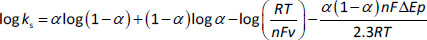



Table 3 shows the different types of modified rGOs as well as the drugs detected and their detection medium, limit of detection (LOD), concentration range and other characteristics.

## Electrodes based on modified screen-printed carbon electrodes for detecting various drugs and metabolites

The screen-printed electrode (SPE) consists of a substrate (PVC, ceramic) onto which miniature electrodes can be printed using ink based on conductive materials. Various substances are employed to increase the sensitivity of the screen-printed electrode, including fullerenes (C60) and iron(III) nanoparticles (Fe_3_O_4_NP).

The antibiotic drug furaltadone (FLT) was detected by a screen-printed carbon electrode (SPCE) decorated with Cu/Ni/TiO_2_/MWCNTs nanocomposites [111]. FLT reduction peak on Cu/Ni/TiO_2_/MWCNTs nanocomposites was located at -470 mV with a significant current density of -39.17 μA. The reduction of the R-NO_2_ group to R-NHOH was performed by electron transfer from HOMO to LUMO in the fabricated electrode and the LOD is equal to 0.0949 μmol l^-1^ with a high sensitivity of 1.9288 μA μmol^-1^ l cm^-2^ in water. We also find that erythronmycin (ERY) is detected by sodium dodecyl sulfate-modified SPCEs (SPCEs/SDS) by flow injection analysis with amperometric detection in PBS of pH 8.5 [112]. Peak detection of ERY is 0.7 V with an LOD of 0.19 μmol l^-1^.

A SPE coated with C60, rGO and Nafion (NF) is used to analysis antibiotic metronidazole (MTZ) in real samples (*cf.* Table 4) [113]. Reduction of GO with NaBH_4_ results in the disappearance of oxygen groups. Fullerenes are used because of their ability to operate at low potentials, which is advantageous for a sensor to avoid interference effects [114,115]. To stabilise the response of the C60-rGO-NF/SPE, the researchers applied two cyclic voltammetry cycles between 0 and -1500 mV at *v* = 20 mV s^-1^ in a 1 mol l^-1^ electrolyte solution of KOH and then the electrode was placed in PBS (Neutre) to apply *v* = 50 mV s^-1^ (scan rate) between 550 and -50 mV. The MTZ reduction peak observed at -0.9 V, representing the exchange of 4e^-^ and 4H^+^. The intensity of the MTZ reduction peak on C60-rGO-NF/SPE is five times higher than that observed on SPE. This sensitivity is explained by the porosity of C60, rGO and NF, which increases the surface area of C60-rGO-NF/SPE. The LOD by SWV is 0.21 μmol l^-1^in the range 0.25-34.0 μmol l^-1^.

GP-CAc/PVC is a screen-printed electrode (SPE) [116], estimated to cost 0.016 $ per unit compared to 7 $ for a commercial electrode, used to determine levodopa (L-dopa) in two commercial drugs, Parkidopa® and Ekson®. These drugs are prescribed to treat Parkinson's disease by reducing the severity of symptoms. To maintain the conductive material of the electrode, researchers have used polymeric materials such as cellulose [117], polyvinyl [118], epoxy glue, nail polish [119] and cellulose acetate (CAc) [120]. In this case, the conductive ink for the electrode is obtained by mixing cellulose acetate and graphite powder in attendance of acetone/cyclohexanone (apolar solvents). The adhesive stencil is used to draw the relief of three electrodes, which are then attached to the PVC. Conductive ink is then applied to the stencil to print the electrodes on the PVC. The stencil is immediately removed to allow the ink to run off. The GP-CAc/PVC has an electroactive surface area of 0.48 cm^2^ with a sensitivity of 0.101 μA μmol^-1^ l owing to the significant porosity of the cellulose acetate. The detection limit by SWV is 0.06 μmol l^-1^ between 8.00 and 100 μmol l^-1^. The researchers found that at pH 5 (pH ≥ 5.0), L-dopa exhibits an irreversible reaction with an oxidation peak located at 0.25 V caused by exchanging the 2H^+^ and 2e^-^. Furthermore, at pH 2 (pH ≥ 4.0), the behaviour of L-dopa changes towards a reversible system with *Ep*(oxy) = 0.27V and *Ep*(red) = -0.01V.

**Figure 5.**
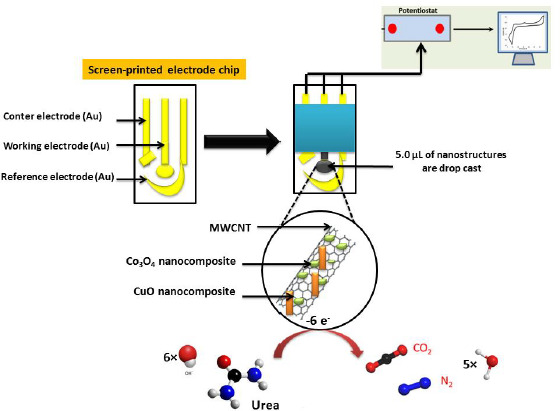
Schematic of CuO/Co_3_O_4_@MWCNTs preparation and electrochemical detection of urea. Based on the reference [121]

On the other hand, SPE modified with the CuO/Co_3_O_4_ nanocomposite integrated MWCNTs [121] has a very low LOD of 0.223 pmol l^-1^ between 10^-12^ and 10^-2^ mol l^-1^ range for urea determination by electrochemical impedance spectroscopy (EIS) (Fig. 5). This performance is due to the combination of CuO and Co_3_O_4_, which have higher electronic conductivity than Co_3_O_4_ or CuO alone.

Table 4 shows the different types of modified SPEs as well as the drugs detected and their detection medium, limit of detection (LOD), concentration range and other characteristics.

## Conclusions and perspectives

This review discusses the different types of recently published electrochemical sensors for the detection of drugs and metabolites in pharmaceutical and biological samples. CPE has a very large electroactive surface area, which can be renewed for a variety of applications. In addition, it has low ohmic resistance and high stability, reproducibility and lifetime, giving it the ability to analyse various drugs, revealing the oxidation and reduction processes involved due to its wide potential range. The sensitivity and speed of analysis of CPE can be significantly improved by modifying them with conductive materials. Various modifiers were used, including organic modifiers like Poly-Eriochrome Black T (Poly-EBT), benzene tricarboxylic acid (BTC), metal-organic frameworks (MOFs) or inorganic modifiers like silver nanoparticles (AgNPs), CuO microfragments (CuO MFs) and TiO_2_. On the other hand, the improvement of CPE can be achieved by using a carbon nanomaterial modifier such as flake graphite (FG), multi-wall carbon nanotubes (MWCNTs) and carbon quantum dots (CQDs). The modified carbon past electrode has been used for the determination of various drugs such as methdilazine hydrochloride (MDH), ketoconazole (KTC), metronidazole (MTZ), sulfamethoxazole (SMZ) in diverse samples such as human urine, pharmaceutical formulations, plasma and serum. Modification with silver nanoparticles is also encouraged due to their biocompatibility, sensitivity, stability and ability to increase peak intensity due to their high conductivity. Similarly, metal oxide nanomaterials such as CuO MFs have an essential contribution to the performance of CPE owing to their wide surface area. L. Hu *et al.* fabricated a selective electrochemiluminescence (ECL) sensor by modifying an CPE with a MIP. The sensor showed a very low LOD of 0.023 nmol l^-1^ to analyse azithromycin (AZI) in real samples.The very low LOD of 0.18 nmol l^-1^ is also found by a CPE modified with lamellar graphite and MWCNT for the analysis of ofloxacin in pharmaceutical tablets and human urine samples. Similarly, CPE modified with Fe_3_O_4_/ZIF-67/ILCPE nanocomposite was applied for the analysis of SMZ in urine and water with a LOD of 5.0 nmol l^-1^.

The transfer rate of electrons at the surface of glassy carbon electrodes (GCE) can be increased by various modifiers such as nitrogen-doped carbon sheets, glucose oxidase, cobalt (II) polymer tetraamino phthalocyanine (polyCoTAPc), methyltrioctyl ammonium chloride (MTOAC) and guanine (ds-DNA). In addition, inorganic modifiers such as iron molybdate (FeMoO_4_), spinel cobalt oxide nanorods (CoCo_2_O_4_ NRs), hexagonal boron nitride (h-BN), cobalt (II) tetra-aminophthalocyanine polymer (polyCoTAPc), cadmium telluride quantum dots (QDs) and titanium carbide (MXene) nanosheets (Ti-C-Tx) have been used for the same purpose. Similarly, a variety of carbon types have been reported in the literature to improve GCE sensitivity, including GO, electrochemically reduced graphene oxide (ERGO), Printex 6L carbon (P6LC), CMK-3 mesoporous carbon (CMK-3), carbon black (CB), graphene (GR), reduced graphene (rGO), graphene-like carbon architecture (HPG) and carboxylic MWCNT (MWCNTseCOOH). The modified GCE was used to determine the drugs in the different matrices. The drugs analysed were: ronidazole, methotrexate, dopamine, amoxicillin, nimesulide, lomefloxacin, sulphamethoxazole, trimethoprim, tryptophan and daunorubicin. Platinum nanoparticles have high electrocatalytic properties. Similarly, DNA nanostructures have high selectivity and affinity for the analyte, which can be used to modify the GCE used for electrochemical drug analysis. The conductivity of the glassy carbon electrodes can be increased by using polymers such as polyaniline (PANI). The combination of various modifiers with high conductivity electrochemical materials such as MIPs, MWCNTs, SWCNTs, ds-DNA and Pt has further increased the sensitivity of the sensors. MIP-MWCNTs/GCE and ds-DNA/Pt/SWCNTs/GCE showed a very low LOD of 1 nmol l^-1^ for drug determination. In addition, the modification with 3D-HPG/PTH shows a similar nanometric detection limit. In 2023, a group of researchers successfully fabricated a glassy carbon electrode modified with CoCo_2_O_4_ NRs/h-BN used to analysis the MTR in pharmaceuticals, with a sensitivity of 5.845 μA μmol^-1^ l cm^-2^ and a LOD of 3 nmol l^-1^.

AuNPs have been employed as modifiers for rGO due to their considerable catalytic activity, biocompatibility and high electrochemical potential, which allows the redox process generated at the electrode surface to be detected. Other materials are employed to improve the sensitivity of rGOs, including glucose oxidase, indium tin oxide (ITO), metal-organic framework (MOF TMU-22) and palladium/ruthenium nanoparticles (Pd-Ru). The modification of rGO with Pd-Ru resulted in a very low LOD of 1.6 nmol l^-1^ for the determination of regorafenib in various samples like human blood, plasma and pharmaceutical formulations.

A variety of conductive compounds are available to increase the sensitivity of the screen-printed electrode, like sodium dodecyl sulphate (SDS), Nafion (NF), fullerene (C60), cellulose acetate, graphite powder, antimony oxide nanoparticles (AONP) and iron (III) nanoparticles (Fe_3_O_4_NP). In addition, multi-wall carbon nanotubes (MWNTs) have a high active surface area and conductivity. The specific structure of these nanomaterials gives them the potential to be an interesting candidate for drug detection electrochemical sensors. The combination of metal oxide and carbon nanotubes significantly increases the sensitivity of SPE. Recently, H. S. Magar *et al.* fabricated an SPE using a combination of CuO/Co_3_O_4_ and MWCNTs. The sensor was used for urea analysis with a very low LOD of 0.223 pmol l^-1^. Not all electrode types mentioned in this paper reported this value. In addition, a LOD of 2.5 nmol l^-1^ was found for nano-Au/MWNTs-ZnO/SPE used to analysis EPI in whole blood and pharmaceutical samples.

The use of nanomaterials and metal oxides seems promising to improve the sensitivity of the electrodes, but there are many problems, such as difficulties in fabrication, handling and characterisation, which encourage investment in finding simpler fabrication methods or extraction from plants and animal secretions.

## Figures and Tables

**Table 1.** Evaluation of electrochemical properties of modified CPE

Electrode	Analyte and matrix	Method	Linear dynamic range	LOD	Year / Ref
poly-EBT/CPE	MDH in Dilosyn syrup and human urine	SWV	0.1-50 μM	0.0257 μM	2020 / [26]
CPE/nanozeolite type X	Paracetamol and epinine	LSV	0.5-70.0 μM	Para = 0.2 μM Epi = 0.008 μM	2023 / [30]
Ce-BTC MOF/IL/CPE	KTC in pharmaceutical and urine samples	Chrono-amperometry; DPV; CV; LSV	0.1-110.0 μM	0.04 μM *S* = 0.1342 μA μM^-1^	2023 / [31]
AgNPs@CPE	MTZ in milk and tap water	-	1-1000 μM	0.206 μM	2022 / [33]
[10%FG/5%MW] CPE	Ofloxacin in pharmaceutical tablets human urine samples	SW-AdAS	0.60 to 15.0 nM	0.18 nM	2019 / [41]
MIP/CP ECL sensor	Azithromycin urine and serum samples	ECL	0.10-400 nM	0.023 nM	2018 / [45]
CPEA/TiO_2_/UV	AA in pharmaceutical tablets	CV	150-850 nM	0.732 μM	2023 / [48]
CuO MFs/Nafion/GC	Glucose in real serum sample	Amperometric	10-120 μM	6.48 μM *S*=3.1 μA μM^-1^ cm^-2^	2018 / [50]
DMIP/CPE	MTZ in Human Serum, Urine and drug	DPV	0.4-200 μM	91 nM	2016 / [52]
Fe_3_O_4_/ZIF-67 /ILCPE	SMZ in urine and water	DPV	0.01-520.0 M	5.0 nM	2021 / [53]

poly-EBT: poly-eriochrome black T; Ce: cerium; BTC: benzene tricarboxylic acid; MOF : metal organic framework; Il : ionic liquids; AgNPs: silver nanoparticles; FG: Flake graphite; MW: multi-wall carbon nanotubes; MIP: molecularly imprinted polymer; ECL: electrochemiluminescence; CuO MFs: CuO micro flowers; DMIP: duplex molecularly imprinted polymer; ZIF-67: zeolitic imidazolate framework ; MDH: methdilazine hydrochloride; KTC: ketoconazole ; AA: ascorbic acid; MTZ: metronidazole; SMZ: sulfamethoxazole; *S*: sensitivity

**Table 2.** Evaluation of electrochemical properties of modified GCE

Electrode	Analyte and matrix	Method	Linear dynamic range	LOD	Year / Ref
GCE/ZnO@NDCS/GOx	Glucose in human blood serum	CV; DPV;CAM	0.2 and 12 mM	6.3 μM *S* = 231.7 μA mM^-1^ cm^-2^	2018 / [54]
FeMoO_4_-GO/GCE	Acyclovir in drug samples	LSV	0.1-10 μM 10-100 μM	20 nM	2022 / [62]
CoCo_2_O_4_ NRs/h-BN/GCE	Ronidazole water samples	DPV	0.01-1345 μM	3 nM *S* = 5.845 μA μM^-1^ cm^-2^	2023 / [67]
GO-Nafion-GCE	MTX in methotrexae injection an uine samples	CV	0.4-20 μM	9 nM	2019 / [73]
GCE-ERGO/polyCoTAPc	DA and Paracetamol in synthetic urine	DPV	-	0.10 μM for PA 0.095 μM for DA *S*_PA_=1.32 μA μM^-1^ cm^-2^ *S*_DA_= 8.39 μA μM^-1^ cm^-2^	2022 / [75]
QDs-P6LC-PEDOT:PSS/GCE	AX in synthetic urine, whole milk and pharmaceuticals	SWV	0.90 to 69 μM	50 nM	2020 / [76]
TiO_2_/CMK-3/AuNPs/Nafion/GCE	AX in pharmaceutical product, mineral and environmental water	CV	LDR_1_= 0.5-2.5 μM LDR_2_ = 2.5-133 μM	0.3 μM *S_1_* = 5071 μA mM^−1^ cm^−2^ *S_2_* = 2971 μA mM^−1^ cm^−2^	2018 / [77]
CB/DPH/GCE	AX inbiological urine, lake and tap water	SWV	2.0-18.8μM	0.12 μM	2018 / [78]
	NIM inbiological urine, lake and tap water	SWV	0.30-5.0 μM	0.016 μM	
AuNPs/PdNPs/ErGO/GCE	AX inhuman urine samples	SWV	30.0-350.0μM	9.0 μM *S* = 0.0376 μA μM^-1^	2017 / [79]
	LMF human urine samples	SWV	4-500 μM	81 nM *S* = 0.0759 μA μM^-1^	
GR-ZnO/GCE	SMZ and TMP in urine and human serum	DPV	LRD_SMZ_= 1-220 μM LRD_TMP_ = 1-180 μM	LOD_SMZ_ = 0.4 μM LRD_TMP_ = 0.3 μM	2020 / [80]
GC/rGO-AgNP	SMZ and TMP in wastewaters samples	DPV	1.0-10.0 mM	LOD_SMZ_ = 0.6 μM LRD_TMP_ = 0.4 μM	2017 / [81]
MIP-MWCNTs/GCE	Trp in the human serum samples.	CV, SDLSVs	2.0 nM-0.2 μM 0.2 -10 μM 10-100 μM	1.0 nM	2020 / [85]
ds-DNA/Pt/SWCNTs/GCE	DRN in daunorubicin injection	DVP	4.0 nM to 250.0 μM	1.0 nM	2021 / [92]
IL-f-ZnONPs@MWCNTs@GCE	SMZ pharmaceutical urine samples	CV	0.1-10 ng ml^-1^	0.1 ng ml^-1^	2022 / [94]
Ag-MWCNT/MTOAC/GCE	SMZ in pharmaceutical formulationsand human urine	DPV	0.05-70 μM	0.01 μM	2018 / [95]
Ti-C-Tx/GCE	AA in human urine samples	CV, DPV	100-1000μM	4.6 μM	2021 / [96]
	DA in human urine samples	CV, DPV	0.5-50 μM	0.06 μM	
	UA in human urine samples	CV, DPV	0.5-4 μM 100-1500 μM	0.075 μM	
PVP-GR/GCE	AA in human urine samples	LSV	4-1000 μM	0.80μM	2020 / [97]
	DA in human urine samples	LSV	0.02-100 μM	0.002 μM	
	UA in human urine samples	LSV	0.04-100 μM	0.02 μM	
rGO-ZnO/GCE	AAin real plasma and urine samples	DPV	50-2350μM	3.71μM	2016 / [98]
	UA in real plasma and urine samples	DPV	1-70 μM	0.33 μM	
	DA in real plasma and urine samples	DPV	3-330 μM	1.08 μM	
3D-HPG/PTH/GCE	MTZ in pharmaceutical and real wate samples	CV; DPV	0.05-70 μM 70−500 μM	1 nM	2018 / [99]
Polydopamine/MWCNTseCOOH nanocomposites/GCE	MTZ in pharmaceutical and biological samples	DPV	5-5000 μM	0.25 μM	2018/ [100]
Ag-MWCNT/MTOAC/GCE	SMZ in pharmaceutical formulations and humanurine	DPV	0.05-70 μM	0.01 μM	2018 / [101]

NDCS: nitrogen-doped carbon sheets; GOx: glucose oxidase; FeMoO_4_-GO: ferrous molybdate-graphene oxide; CoCo_2_O_4_ NRs: spinel cobalt oxide nanorods; h-BN: hexagonal boron nitride; ERGO: electrochemically reduced graphene oxide; polyCoTAPc: polymer of cobalt (II) tetra-amino phthalocyanine; QDs: cadmium telluride quantum dots; P6LC: Printex 6L Carbon; PEDOT: poly(3,4-ethylenedioxythiophene; PSS: polystyrene sulphonate film; CMK-3: mesoporous carbon CMK-3, AuNPs: gold nanoparticles; DPH: dihexadecyl hydrogen phosphate; CB: carbon black; PdNPs: palladium nanoparticles; GR: graphene; MIP: molecularly imprinted polymer; ds-DNA: guanine-based; IL-f-ZnONPs: ionic liquid functionalised zinc oxide nanoparticles; MTOAC: methyltrioctyl ammonium chloride; Ti-C-Tx: titanium carbide (MXene) nanosheets; PVP: polyvinylpyrrolidone; rGO: Reduced graphene; 3D: three-dimensional; HPG: graphene-like carbon architecture; PTH: polythionine; MWCNTseCOOH: carboxylic MWCNT; RNZ: ronidazole; MTX: methotrexate; DA: dopamine; AX: Amoxicillin, NIM: nimesulide; LMF: lomefloxacin; SMZ: sulfamethoxazole; TMP: trimethoprim; Trp: tryptophan; DNR: daunorubicin, AA: acide ascoorbic; DA: dopamine; UA: uric acid; MT^Z^: metronidazole; *S*: sensitivity; CAM: chronoamperometry.

**Table 3.** Evaluation of electrochemical properties of modified rGO

Electrode	Analyte and matrix	Method	Linear dynamic range	LOD	Other characteristics	Year/Ref.
Nafion/GOx/Au-ZnO/rGO/ITO	glucose in blood	Amperometric	0-9.5 mM	0.2 μM	*S* = 10.93 μA mM^-1^ cm^-2^	2020 / [104]
Pd-Ru/rGO	Regorafenib in human blood and plasma pharmaceutical formulation	DPV	0.5-300 nM	1.6 nM	*k*_S_ = 2.29 s^-1^	2022 / [108]
rGO/TMU-22 MOF	Levodopa in human urine and tablet samples	CV; SWV	0.1-85 μM 0.1-85 μM	25 nM	*S* = 0.58 μA μM^−1^ *k*_S_ = 7.7 s^−1^	2020 / [110]

AuNPs : gold nanoparticles ; GOx: glucose oxidase; rGO: reduced graphene oxide;ITO: indium tin oxide ; TMU-22 MOF: metal-organic framework; Pd-Ru: palladium/ruthenium nanoparticles; *S*: sensitivity

**Table 4.** Evaluation of electrochemical properties of modified SPE

Electrode	Analyte and matrix	Method	Linear dynamic range	LOD / Other characteristics	Year / Ref.
Cu/Ni/TiO_2_/MWCNTs/SPCE	FLT in water	CV; DPV	10-150 μM	0.0949 μM *S* = 1.9288 μA μM^-1^ cm^-2^	2022 / [111]
SPCEs/SDS	ERY in water	Amperometric	1-15 mg l^-1^	0.19 μM	2019 / [112]
C60-rGO-NF/SPE	MTZ in serum and urine samples	SWV	0.25-34.0 μM	0.21 μM	2021 / [113]
GP-CAc/PVC	Levodopa in two commercial drugs	SWV	8-100 μM	0.06 μM	2021 / [116]
CuO/Co3O4@MWCNTs/SPE	Urea	EIS	10^-12^-10^-2^ M	0.223 pM	2023 / [121]
Nano-Au/MWNTs-ZnO/SPE	MTX in whole blood samples and pharmaceutical	SWV	0.02-1.00 μM	10 nM	2014 / [122]
	EPI in whole blood samples and pharmaceutical	SWV	0.005-0.2 μM	2.5 nM	
MWCNT-AONP/SPCE	AA in fresh oranges	SWV	160-640 nM	140 nM	2022 / [123]
SPCE/CV-Fe_3_O_4_NP	AA	SWV	10-100 μM	15.7μM	2021 / [124]

SDS: sodium dodecyl sulfate ; NF: Nafion; C60: fullerene ; CAc: cellulose acetate; GP: graphite powder; PVC: polyvinyl chloride; AONP: antimony oxide nanoparticle; CV: Callistemon viminalis; Fe_3_O_4_NP: Iron(III) nanoparticle; FLT: furaltadone; ERY:erythromycin; MTZ:metronidazole ; MTX : methotrexate; EPI : epirubicin; AA: ascorbic acid.

## List of abbreviations

C60: Buckminsterfullerenes
CA: Chronoamperometry
CPE: Carbon paste electrodes
CPEA: Carbon clay paste electrode
CV: Cyclic voltammetry
DPV: Differential pulse voltammetry
EBT: Eriochrome black T
ECL: Electrochemiluminescence
EIS: Electrochemical impedance spectroscopy
ERGO: Electrochemically reduced graphene oxide
FG: Flake graphite
FTIR: Fourier transform infrared
GCE: Glassy carbon electrode
GO: Graphene oxide
GP: Graphite powder
GR: Graphene
HOMO: Highest occupied molecular orbital
HPLC-DAD: High performance liquid chromatography-diode array detector
LOD: Limit of detection
LSV: Linear scan voltammetry
LUMO: Lowest unoccupied molecular orbital
MIP: Molecularly imprinted polymer
MWCNTs: Multi-wall carbon nanotubes
MXene: M: transition metal, X: carbon and/or nitrogen, ene: similarity to graphene
NF: Nafion
PBS: Phosphate-buffered saline
PVC: Polyvinyl chloride
rGO: Reduced graphene oxide
SDLSV: Second-order derivative linear sweep voltammetry
SPC: Screen-printed carbon
SPCE: Screen-printed carbon electrode
SW-AdAS: Square wave adsorptive anodic stripping voltammetry
SWCNT: Single-walled carbon nanotubes
SWV: Square wave voltammetry
TLC-densitometric: Thin layer chromatography densitometric
